# Explainable machine learning using pretreatment complete blood count parameters and demographic characteristics for gastric cancer risk stratification: a temporal validation study

**DOI:** 10.3389/fimmu.2026.1892111

**Published:** 2026-10-15

**Authors:** Yongfeng Qiao, Qian Long, Jin Tang, Xin Wang, Shouli Wang

**Affiliations:** 1 Department of Clinical Laboratory, Hanzhong Central Hospital, Hanzhong, Shaanxi, China; 2 Department of Pathology, Hanzhong Central Hospital, Hanzhong, Shaanxi, China; 3 Department of Surgical Oncology, Hanzhong Central Hospital, Hanzhong, Shaanxi, China

**Keywords:** complete blood count, explainable artificial intelligence, gastric cancer, machine learning, risk stratification, SHAP, temporal validation, tumour markers

## Abstract

**Background:**

Gastric cancer (GC) is often diagnosed at an advanced stage, while endoscopy is invasive and conventional serum tumour markers have limited sensitivity. A low-cost, widely available tool that can identify patients at higher risk and prioritise them for endoscopic evaluation could therefore strengthen early-detection pathways. The complete blood count (CBC) is routinely available and well suited to such first-contact risk stratification; however, existing CBC-based machine-learning models are often developed in cohorts that mix prevalent, postoperative, on-treatment and surveillance records, potentially confounding the haematological signal. We therefore developed and temporally validated an explainable CBC-based model to stratify the risk of newly diagnosed GC versus benign gastric disease in a rigorously verified, treatment-naïve cohort.

**Methods:**

In this retrospective single-centre study, 2,044 patients (477 pathologically confirmed, newly diagnosed, treatment-naïve GC and 1,567 benign gastric-disease controls) were assembled through case-by-case chart review, patient-level identity de-duplication and application of the same prespecified comorbidity-screening criteria. The baseline CBC was obtained at admission before treatment. A parsimonious model incorporated six routinely available variables: age, sex, and neutrophil, lymphocyte, monocyte and platelet counts. Eight algorithms were trained using five-fold cross-validation in the training cohort (n = 1,309) and evaluated in a temporally separated validation cohort (n = 735). Random forest was designated as the reference explainable model and interpreted using SHAP. Model behaviour in tumour-marker-negative GC and robustness in sensitivity analyses were also assessed.

**Results:**

Discrimination was similar across algorithms. In temporal validation, random forest achieved an AUC of 0.831 (95% CI 0.798–0.864), which was numerically similar to that of the highest-AUC model, XGBoost (AUC 0.832), with substantially overlapping 95% confidence intervals. Random forest had a low Brier score (0.133) and showed positive net benefit across the evaluated threshold-probability range. SHAP identified lymphocyte count, male sex and age as the leading predictors, with an identical feature ranking under XGBoost (Spearman ρ = 1.00). Adding derived inflammatory ratios did not improve discrimination (ΔAUC −0.010, p = 0.139). Among temporal-validation patients with triple-negative GC, defined as CEA-, CA19-9- and CA72-4-negative disease, the model classified 62.7% (95% CI 50.0–73.9) as high risk.

**Conclusions:**

A six-variable model combining pretreatment CBC parameters with age and sex discriminated newly diagnosed GC from benign gastric disease in a temporally separated validation cohort and showed broadly acceptable graphical calibration and a low Brier score. The model classified a substantial proportion of tumour-marker-negative GC cases as high risk, supporting further evaluation as a low-cost pre-endoscopic triage aid. External multicentre validation is required.

## Introduction

Gastric cancer (GC) remains among the leading causes of cancer death worldwide, and outcome is strongly stage-dependent: disease detected early is potentially curable, whereas the majority of cases, diagnosed once symptomatic, carry a poor prognosis. Endoscopy with biopsy is the diagnostic reference standard but is invasive, resource-intensive and impractical as a first-line triage tool for unselected or low-risk populations. Serum tumour markers such as CEA, CA19–9 and CA72–4 have limited and variable sensitivity for GC, particularly early disease; pepsinogen-based biomarkers and precursor-lesion surveillance strategies may enrich risk in selected settings but do not provide a universally available first-contact triage test. There is therefore a need for inexpensive, routinely available tools that can flag individuals warranting endoscopic evaluation (1–15).

The complete blood count (CBC) is the most frequently performed laboratory test in routine practice, and peripheral-blood immune–inflammatory signatures — reflected in cell counts and their derived ratios (for example the neutrophil-to-lymphocyte, platelet-to-lymphocyte and lymphocyte-to-monocyte ratios, and composite indices such as the systemic immune-inflammation index) — have been associated with malignancy. Machine-learning models built on such parameters have accordingly been proposed for GC risk stratification. However, the interpretability and clinical credibility of these models are undermined by two recurrent limitations of their development cohorts. First, cohort labels are often assembled from structured diagnosis fields without case-level verification, so that prevalent, post-operative, on-treatment or surveillance records are mixed with genuinely new diagnoses; the learned haematological signal may then be contaminated by treatment- and comorbidity-related perturbation. Second, feature sets frequently combine raw counts with the composite ratios derived from them, introducing deterministic collinearity that obscures which quantities actually carry information (15–33).

More broadly, recent advances in artificial-intelligence-assisted precision medicine show how machine-learning methods can recover nonlinear and multidimensional patterns from routine clinical variables, imaging and omics data and translate them into clinically useful risk signatures. In gastric cancer, recent work has repurposed routine blood tests for opportunistic endoscopy triage, combined CT radiomics with clinical information for preoperative risk stratification, and integrated spatial and transcriptomic information with deep learning for prognostic phenotyping (15, 34, 35). Beyond oncology, machine-learning integration with metabolomic profiling and chemical-structure-aware graph learning illustrates how complex biological measurements can be distilled into diagnostic or mechanistically informative signatures (36, 37). These developments support the use of machine learning not simply to increase model complexity, but to identify reproducible patterns that can be examined for biological and clinical plausibility.

We addressed both limitations directly. The study cohort was restricted to pathologically confirmed, newly diagnosed, treatment-naïve GC, with the baseline CBC drawn at admission (day 0–1) before any anti-tumour treatment; eligibility, treatment-naïve status and comorbidities were verified case-by-case by two independent reviewers, and identical comorbidity screening was applied to benign controls, with patient-level de-duplication by Chinese national resident identity card number. This design reduces the risk of contamination from treatment-related haematological changes and supports more specific characterisation of a disease-associated peripheral-blood pattern, while providing a label-quality foundation consistent with the TRIPOD+AI reporting framework — positioning label integrity, rather than algorithmic novelty alone, as a principal contribution. Within this cohort we developed a deliberately parsimonious model using only six routinely measured variables (age, sex, and the neutrophil, lymphocyte, monocyte and platelet counts), avoiding redundant derived ratios in the primary specification; temporally validated it; interpreted it with SHAP and confirmed the stability of the explanation across model families; and characterised model recall in the clinically important subgroup of tumour-marker-negative GC (38, 39).

## Methods

### Study design and data source

This was a retrospective, single-centre diagnostic modelling study based on routine laboratory records extracted from the hospital and laboratory information systems (HIS/LIS), together with case-by-case review of the corresponding clinical charts. The objective was to develop and temporally validate an explainable machine-learning model that discriminates newly diagnosed, treatment-naïve gastric cancer (GC) from benign gastric disease using only routinely available complete blood count (CBC) parameters together with age and sex, and to characterise the behaviour of the reference model in tumour-marker-negative GC. The study is reported in accordance with the TRIPOD+AI statement for prediction-model studies and informed by diagnostic-accuracy reporting guidance (38–41).

### Ethical approval

The study was approved by the institutional ethics committee (Approval No. 2026-LW-09), which waived the requirement for individual informed consent given the retrospective use of de-identified records. Patient identifiers (name and Chinese national resident identity card number) were used solely for record linkage and were removed before analysis; no identifiers entered the modelling datasets.

### Study population and cohort definitions

#### Gastric cancer cohort (pathologically confirmed, newly diagnosed, treatment-naïve)

GC cases were required to satisfy all of the following criteria, verified by chart review:

- Histopathological confirmation of a primary gastric malignancy from an endoscopic biopsy or surgical specimen obtained at the study centre.
- Newly diagnosed disease at the study centre, with the date of first histopathological confirmation taken as the index date. Patients with any prior GC diagnosis, or who had been diagnosed and/or treated at an external institution before presentation, were excluded on case-by-case review of the clinical course, presenting complaint and past history.
- Treatment-naïve status at sampling. The baseline CBC used for modelling was drawn at admission (day 0–1) and, in every case, before any anti-tumour intervention (surgery, chemotherapy or radiotherapy). For patients whose diagnosis was ultimately confirmed on a surgical specimen, the modelling CBC was the pre-operative admission sample; post-treatment samples were never used.

Records explicitly indicating non-index status (post-operative state, follow-up or surveillance review, or historical/external records) were not selected as index records.

#### Benign comparator cohort

Benign cases comprised patients with a clinician-assigned diagnosis of benign gastric disease (chronic, superficial, erosive, reflux, bile-reflux or verrucous gastritis; gastric polyps; or gastric ulcer with or without bleeding). For inpatients, the clinical course was reviewed on a case-by-case basis using the same procedure as for the GC cohort; for outpatients, all records under the patient’s outpatient identifier were reviewed. Benign patients typically underwent phlebotomy at the index presentation without prior intervention, consistent with the usual management of benign gastric disease. Any patient found on review to have a GC diagnosis at any point was not classified as benign.

#### Comorbidity screening (applied identically to both cohorts)

Exclusionary comorbidities were defined *a priori* and applied identically to both cohorts. Patients were excluded only when the condition was documented before or at the index encounter, or when chart review indicated that it was active at baseline. Prespecified exclusions were active infection; primary haematological malignancy or other major haematological disorder; clinically diagnosed leucopenia or cytopenia unrelated to GC; and chronic kidney disease requiring dialysis. Leucopenia/cytopenia exclusions were based on clinical diagnoses or documented medical history, not on the CBC values used as model predictors, and an abnormal baseline CBC value alone was not an exclusion criterion. Diagnoses first recorded after the index date were not used for exclusion unless the clinical record showed that the disorder was already present at baseline. Common chronic comorbidities, including diabetes mellitus, were not prespecified exclusions and were deliberately retained to preserve real-world applicability; their potential residual influence on CBC measurements is considered in the Discussion (16, 17).

#### Record linkage and label review

Patient-level identity linkage and de-duplication were performed using the Chinese national resident identity card number, ensuring that each patient contributed a single baseline record and that no patient appeared in more than one analytic group. All eligibility, index-date, treatment-naïve and comorbidity determinations were made by two independent reviewers through manual inspection of the pathology reports and clinical records; discrepancies were resolved by discussion. Accordingly, all retained GC cases were confirmed to have no previous history of GC and to have received no anti-tumour treatment before the index admission.

#### Cohort derivation and the analytic dataset

Candidate patients were assembled from HIS/LIS records by diagnosis-keyword screening (795 GC and 2,586 benign candidates; **Supplementary Table 1**) and resolved by patient-level identity adjudication using the Chinese national resident identity card number, which removed records without a complete identity card number (GC n = 32; benign n = 201) and cross-group overlaps (20 overlapping patients, corresponding to 40 group-specific candidate entries), yielding 3,108 adjudicated patients (743 GC, 2,365 benign). Each cohort was then reduced to its model-ready analytic set through eligibility, baseline-CBC and comorbidity review. In the GC pathway, 266 patients were excluded (prior GC before the study window, n = 175; outpatient-coded GC without evidence of recent onset, n = 86; no usable baseline CBC, n = 5), leaving 477 GC. After application of these exclusions, no remaining GC candidates met the prespecified comorbidity exclusion criteria. In the benign pathway, 798 patients were excluded (age < 18 years, n = 50; no eligible benign diagnosis, n = 21; no usable baseline CBC, n = 441; gastritis not the primary inpatient diagnosis, n = 187; and prespecified CBC-altering conditions on outpatient review, including major haematological disease, clinically diagnosed leucopenia/cytopenia or dialysis-dependent chronic kidney disease, n = 99), leaving 1,567 benign. The resulting analytic cohort comprised 2,044 patients (477 GC, 1,567 benign). The full derivation, including the diagnosis-keyword rules, is shown in **Figure 1**; **Supplementary Table 1** (38, 39).

**Figure 1:**
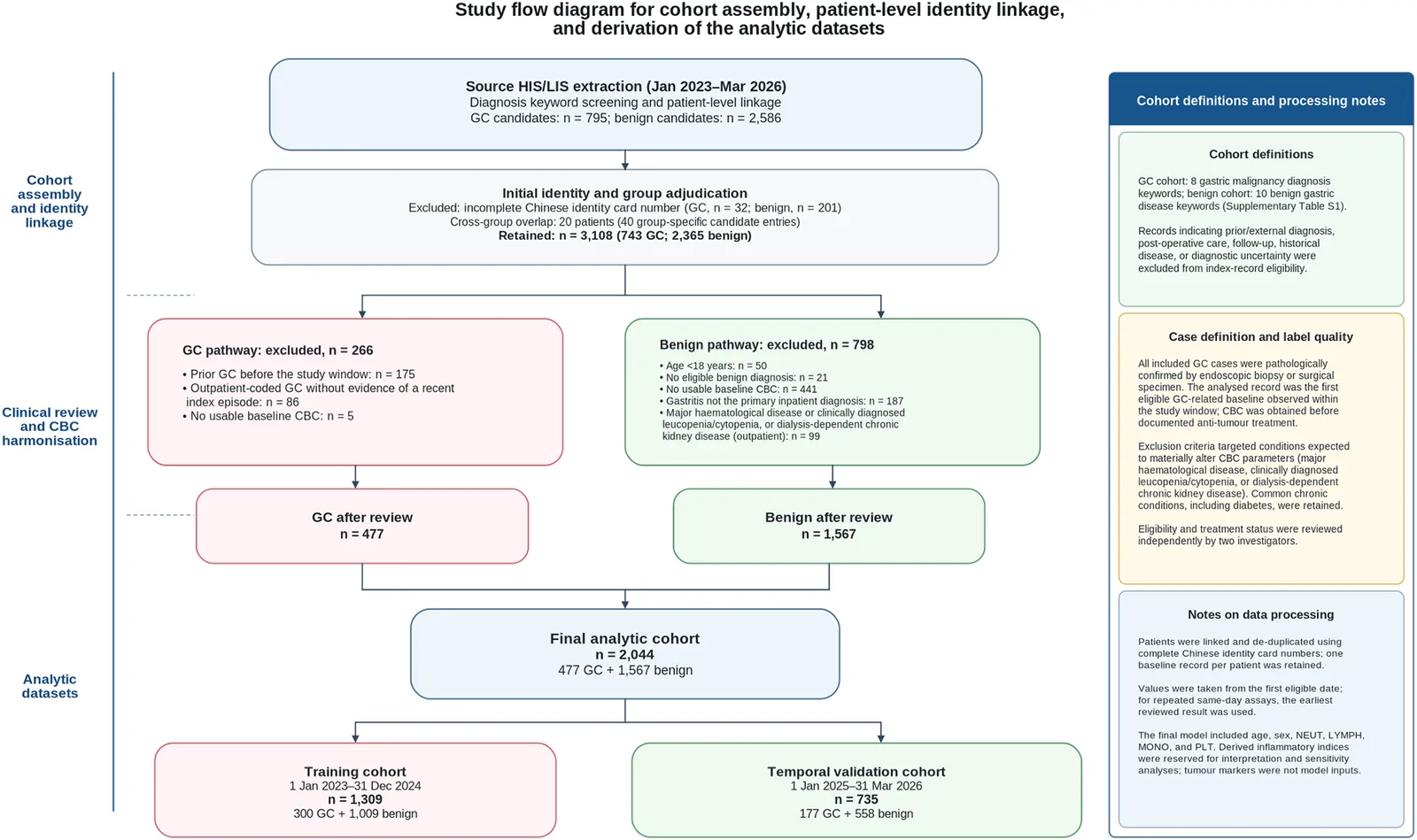
Study flow diagram for cohort assembly, patient-level identity linkage, and derivation of the analytic datasets. Diagnosis-keyword screening and patient-level identity adjudication based on Chinese national resident identity card numbers yielded 3,108 adjudicated patients; parallel gastric cancer (GC) and benign pathways involving eligibility, baseline complete blood count (CBC), and comorbidity review produced the final analytic cohort of 2,044 patients (477 GC and 1,567 benign), which was partitioned by baseline date into training (n = 1,309) and temporal validation (n = 735) cohorts. Diagnosis-keyword rules are given in **Supplementary Table 1**.

### Outcome

The binary outcome was newly diagnosed, treatment-naïve GC (index record; coded 1) versus benign gastric disease (coded 0).

### Candidate predictors

#### Primary predictor set

The primary model used six routinely available variables: age, sex, and absolute neutrophil (NEUT), lymphocyte (LYMPH), monocyte (MONO) and platelet (PLT) counts, all derived from a single admission CBC obtained before treatment. This parsimonious specification was selected because (i) the inputs are available at first clinical contact in many healthcare settings; (ii) restricting the model to directly measured quantities avoids the deterministic collinearity introduced when composite ratios are entered alongside their constituent variables; and (iii) tree-based learners can model nonlinear effects and interactions among the component counts without explicitly constructing derived ratios.

#### Derived inflammatory indices (used only for interpretation and sensitivity analysis)

Five composite indices were computed from the raw counts for interpretive and robustness purposes only: NLR = NEUT/LYMPH, PLR = PLT/LYMPH, LMR = LYMPH/MONO, SII = PLT × NEUT/LYMPH and SIRI = NEUT × MONO/LYMPH. Because each is a deterministic function of the primary counts, these indices were not entered into the primary model; they were used to interpret the model in immunological terms and to test an alternative, ratio-augmented feature representation (see Sensitivity analyses) (20–24).

#### Tumour markers were not model inputs

Serum tumour markers (CEA, CA19-9, CA72-4, AFP, CA125) were available for a subset of GC cases but were not systematically available in the benign cohort, reflecting routine test-ordering practices. Including their availability or values as model inputs could therefore confound disease status with test-ordering patterns and create artefactual separation between the groups. Tumour markers were consequently excluded from all model inputs and were used exclusively for a *post hoc* description of model recall among tumour-marker-negative GC cases (see below).

### Missing data

Consistent with the baseline-CBC eligibility applied during cohort derivation, the six primary predictors were complete in the analytic cohort (no missing values); modelling therefore used complete cases without imputation. The principal missing-data issue occurred before model entry: absence of a usable baseline CBC was rare among adjudicated GC candidates (5/743, 0.7%) but common among adjudicated benign candidates (441/2,365, 18.6%), reflecting that many benign gastric-disease encounters, especially outpatient visits, did not have a contemporaneous CBC suitable for baseline modelling. We did not use multiple imputation for these patients because the core CBC measurements were unavailable or not eligible as a baseline sample; imputing them would create synthetic laboratory profiles rather than recover observed triage-test results. This differential availability was therefore handled by transparent exclusion and is acknowledged as a potential source of selection bias.

### Sample size considerations

As this was a retrospective study, the sample size was determined by the number of eligible patients available during the study period. The overall analytic cohort comprised 2,044 patients (477 GC, 1,567 benign; GC proportion 23.3%), of whom 1,309 patients (300 GC, 1,009 benign) formed the model-development training cohort and 735 patients (177 GC, 558 benign) formed the temporal validation cohort. Model-development adequacy was assessed pragmatically on the basis of 300 GC events for six prespecified predictors, the parsimonious model specification, cross-validated performance stability and the precision of temporal-validation estimates. The events-per-predictor ratio is reported descriptively and was not treated as a formal power calculation, consistent with contemporary prediction-model sample-size guidance (42).

### Data partitioning

The overall analytic cohort was partitioned *a priori* by baseline-record date into a derivation (training) cohort of 1,309 patients (300 GC, 1,009 benign; baseline dates 1 January 2023 to 31 December 2024) and a temporally separated validation cohort of 735 patients (177 GC, 558 benign; baseline dates 1 January 2025 to 31 March 2026). The temporal validation cohort was withheld from feature handling, model fitting, hyperparameter tuning and threshold selection.

### Data preprocessing and leakage control

All preprocessing was fitted on the training data only and applied unchanged to the temporal validation cohort. For scale-sensitive learners (logistic regression, support-vector machine, k-nearest neighbours, naïve Bayes), predictors were standardised using training-set means and standard deviations; tree-based ensembles were fitted on the raw predictors. No transformation, resampling or threshold decision used information from the validation cohort.

### Model development

Eight algorithms spanning linear, kernel, instance-based, probabilistic and ensemble families were trained: logistic regression, naïve Bayes, k-nearest neighbours, decision tree, random forest, support-vector machine, extreme gradient boosting (XGBoost) and light gradient-boosting machine (LightGBM). Hyperparameters were tuned within the training set by five-fold cross-validation optimising the area under the receiver-operating-characteristic curve (AUC), using the grids and selected settings listed in **Supplementary Table 9**. Cross-validated discrimination was summarised as the mean ± standard deviation of the fold-wise AUCs and pooled out-of-fold predictions were used for the training-set ROC display. The random forest was designated as the reference explainable model on methodological and interpretability grounds, particularly its compatibility with exact tree-based SHAP analysis. The temporal validation set was not used to optimise features, hyperparameters or thresholds, and all eight algorithms are reported for benchmarking (25–33, 38, 43).

### Class imbalance

GC accounted for 23.3% of the analytic cohort. Synthetic oversampling was not used. Class weighting was evaluated in a sensitivity analysis, and no *post-hoc* probability recalibration was applied. Operating thresholds were selected using the training data only.

### Model evaluation

Performance in the temporal validation cohort was assessed across three complementary dimensions: discrimination (AUC with 95% confidence intervals by the DeLong method), calibration/probabilistic accuracy (Brier score and calibration curves of observed versus mean predicted probability using 10 quantile bins) and clinical utility (decision-curve analysis of net benefit across an evaluated range of threshold probabilities, referenced against treat-all and treat-none). A single operating threshold was derived in the training set at the maximum Youden index and applied unchanged to the temporal validation cohort. At this threshold, and at the fixed thresholds 0.30, 0.40 and 0.50, sensitivity, specificity, positive and negative predictive value, F1 score, Matthews correlation coefficient and the predicted-positive proportion were reported (44–46).

### Model explainability

The primary random-forest model was interpreted with SHapley Additive exPlanations (SHAP) using the exact tree estimator. Global importance was summarised as the mean absolute SHAP value per predictor; a beeswarm summary and per-feature dependence plots described the direction and shape of each contribution; and individual-level SHAP contribution plots illustrated representative correctly classified GC and benign cases from the temporal validation cohort. Because the CBC was uniformly obtained before anti-tumour treatment and major CBC-altering comorbidities were excluded, the recovered contributions were interpreted as a disease-associated peripheral-blood pattern with reduced contamination from treatment- or comorbidity-related perturbation, rather than as evidence of a causal mechanism. Global, dependence and individual SHAP outputs are shown in **Figures 2**–**4**, and global importance values are tabulated in **Supplementary Table 5** (47).

**Figure 2:**
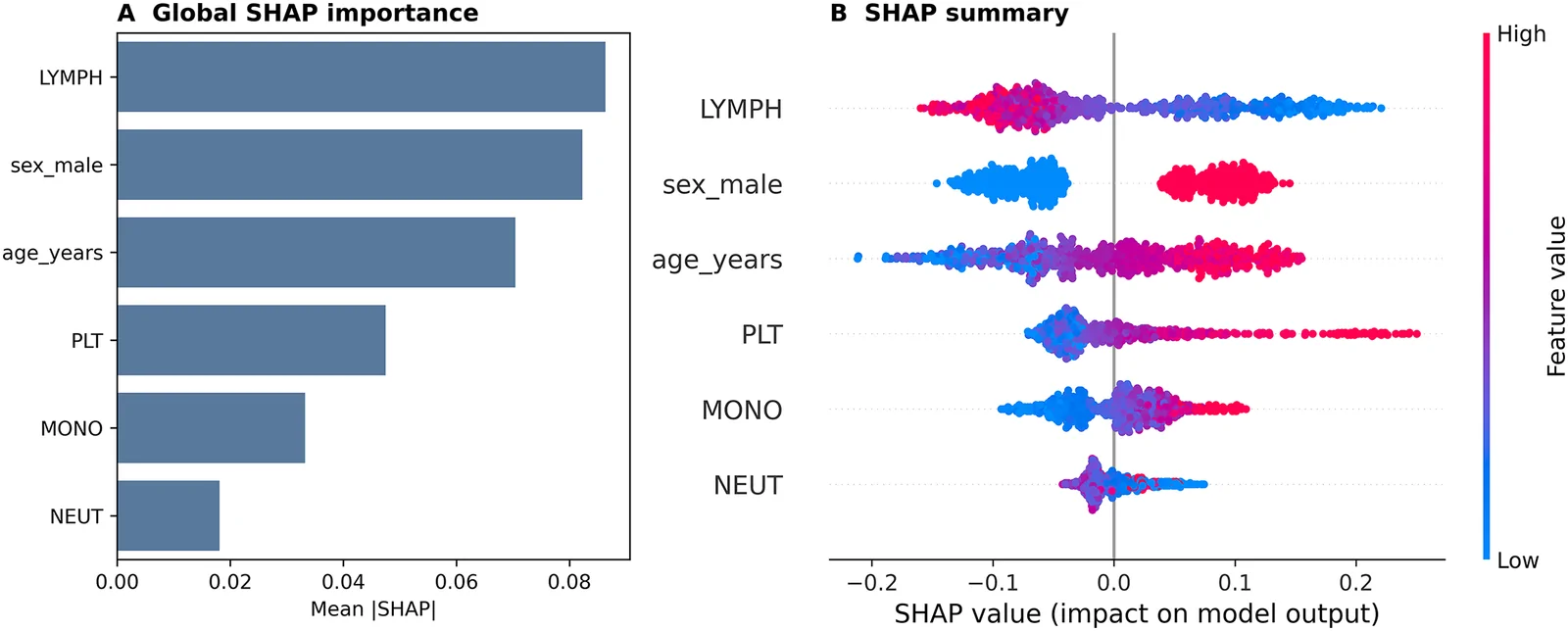
Global SHAP interpretation of the primary random-forest model. **(A)** Global feature importance, quantified as the mean absolute SHAP value for each predictor. **(B)** SHAP summary plot showing the distribution, direction and magnitude of each predictor’s contribution to the model output. Each point represents one patient, and the colour indicates the corresponding feature value, ranging from low (blue) to high (red). Positive SHAP values increase the predicted probability of gastric cancer, whereas negative SHAP values decrease it.

**Figure 3:**
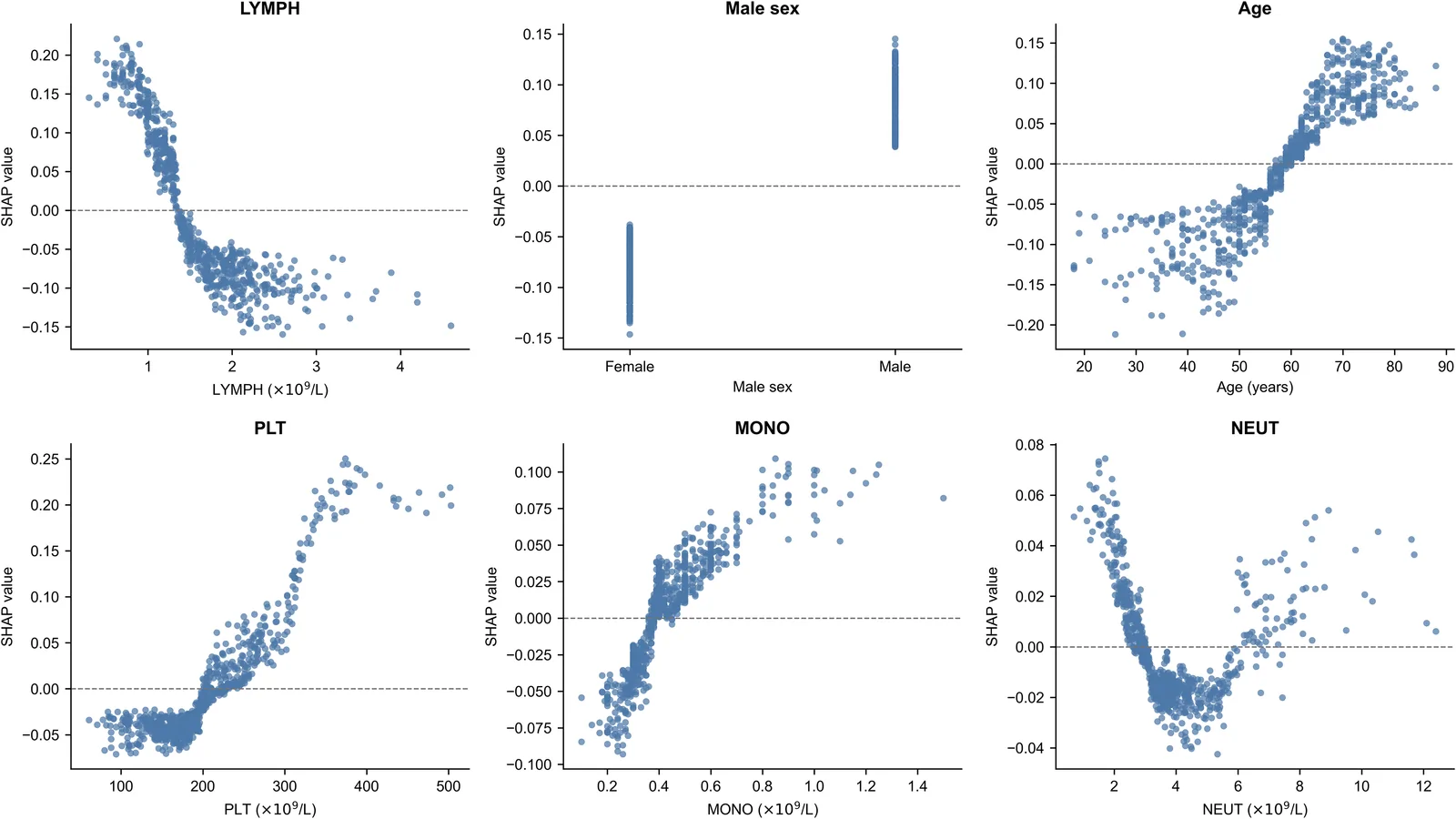
SHAP dependence plots for the six predictors in the primary random-forest model. Each point represents one patient and shows the relationship between the original predictor value and its SHAP contribution to the model output. Positive SHAP values increase the predicted probability of gastric cancer, whereas negative SHAP values decrease it. The dashed horizontal line denotes a SHAP value of zero.

**Figure 4:**
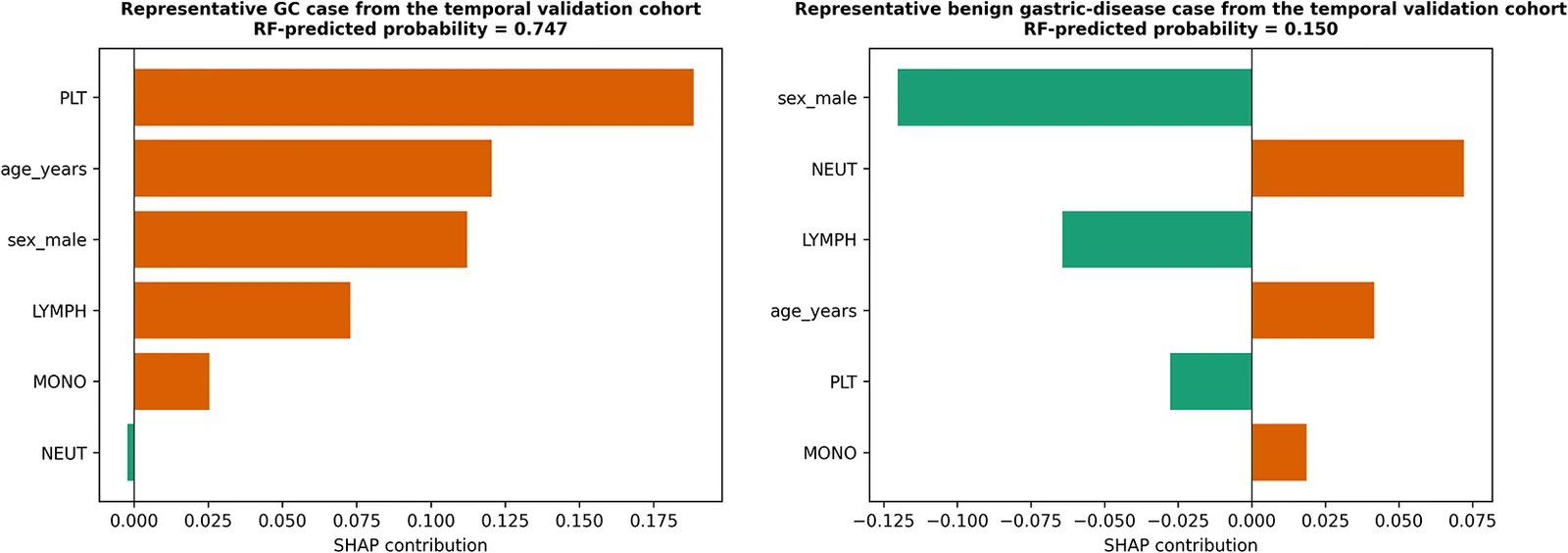
Local SHAP explanations for representative gastric cancer and benign gastric-disease cases from the temporal validation cohort. Bars represent feature-level SHAP contributions to the primary random-forest model output. Positive SHAP contributions (orange) increase the predicted probability of gastric cancer (GC), whereas negative SHAP contributions (green) decrease it. The displayed probability reflects the model baseline value plus the sum of feature-level SHAP contributions.

### Tumour-marker-negative GC subgroup

To characterise model behaviour in GC cases not positive for conventional serum markers, a subgroup analysis was performed within the GC cohort. Among GC patients in whom CEA, CA19–9 and CA72–4 had all been measured, a triple-negative subgroup was defined as those with all three markers below the institutional reference thresholds (CEA < 5 ng/mL, CA19-9 < 37 U/mL, CA72-4 < 6.9 U/mL; censored high values were treated as above threshold). The proportion of these tumour-marker-negative GC cases that the model flagged high-risk (at the training-derived Youden threshold and at 0.30, 0.40 and 0.50) was reported as within-GC recall, with Wilson 95% confidence intervals. To avoid in-sample optimism, this analysis was performed primarily in the temporal validation cohort (out-of-sample); the corresponding whole-GC-cohort estimates are reported descriptively in **Supplementary Table 8**. Because the benign cohort lacked marker data, this analysis characterises within-GC sensitivity among marker-negative cases and does not constitute a head-to-head comparison of the model against tumour markers.

### Sensitivity analyses

Robustness was examined by (i) refitting under an alternative, ratio-augmented feature representation adding NLR, PLR, LMR, SII and SIRI to the primary counts, with multicollinearity quantified by the variance inflation factor before and after LASSO selection (**Supplementary Table 2**), and discrimination compared with the primary model (**Supplementary Table 3**); (ii) evaluating class weighting as an alternative to the unweighted model (**Supplementary Table 4**); (iii) repeating the operating-characteristic analysis across the fixed thresholds above (**Supplementary Table 6**); and (iv) assessing the stability of the explanations by comparing global SHAP importance between the primary random forest and an XGBoost model, quantified by the Spearman rank correlation of mean absolute SHAP values (**Supplementary Table 5** and **Supplementary Figure 2**; feature correlations are shown in **Supplementary Figure 1**). Discrimination differences between nested feature sets were tested with the DeLong test for correlated AUCs (44, 47, 48).

### Statistical software and reproducibility

Analyses were performed in Python version 3.14 with scikit-learn, XGBoost, LightGBM, SHAP, statsmodels, pandas and NumPy. Random seeds were fixed for all stochastic procedures, and the analysis code is available from the corresponding author upon reasonable request. Continuous variables are summarised as median (interquartile range) and categorical variables as counts (percentages); two-sided p < 0.05 was considered statistically significant.

## Results

### Cohort characteristics

The analytic cohort comprised 2,044 patients — 477 with newly diagnosed, treatment-naïve gastric cancer (GC) and 1,567 with benign gastric disease (GC proportion 23.3%) — partitioned *a priori* by baseline date into a training set of 1,309 patients (300 GC, 1,009 benign) and a temporally separated validation set of 735 patients (177 GC, 558 benign). Compared with benign controls, patients with GC were older (median 66 vs 56–57 years) and more often male (approximately 74% vs 38–40%), and showed a systematically more inflammatory peripheral-blood profile: lower lymphocyte counts and lymphocyte-to-monocyte ratio, and higher monocyte and platelet counts, neutrophil-to-lymphocyte ratio, platelet-to-lymphocyte ratio, systemic immune-inflammation index and systemic inflammation response index (all p < 0.001). Neutrophil counts did not differ significantly between groups (p = 0.076). Baseline characteristics are summarised in **Table 1**.

**Table 1:** Baseline demographic and haematological characteristics of the study cohorts.

Variable	Training cohort		Temporal validation cohort		*p* ^a^
	Gastric cancer (n = 300)	Benign control (n = 1,009)	Gastric cancer(n = 177)	Benign control (n = 558)	
Age, y	66 (58-72)	57 (49-65)	66 (59-73)	56 (48-63)	<0.001
Male sex	223 (74.3)	403 (39.9)	132 (74.6)	213 (38.2)	<0.001
Haematological indices^b^					
NEUT (10^9/L) ^b^	3.62 (2.58-4.91)	3.43 (2.69-4.40)	3.72 (2.99-5.09)	3.50 (2.71-4.30)	0.076
LYMPH (10^9/L)	1.21 (0.91-1.54)	1.60 (1.26-1.97)	1.25 (0.94-1.68)	1.59 (1.21-2.00)	<0.001
MONO (10^9/L)	0.45 (0.36-0.58)	0.38 (0.30-0.49)	0.44 (0.37-0.56)	0.39 (0.30-0.47)	<0.001
PLT (10^9/L)	210.50 (162.00-266.25)	192.00 (159.00-235.00)	205.00 (166.00-272.00)	194.00 (159.00-231.00)	<0.001
Inflammatory indices^c^					
NLR	2.99 (1.99-4.47)	2.09 (1.56-2.98)	3.08 (2.04-4.60)	2.16 (1.53-3.08)	<0.001
PLR	167.59 (126.27-235.84)	120.00 (92.64-154.20)	164.21 (115.00-264.47)	119.25 (94.65-159.51)	<0.001
LMR	2.67 (1.95-3.67)	4.32 (3.22-5.44)	3.00 (2.04-3.90)	4.25 (3.23-5.23)	<0.001
SII	612.29 (372.34-1063.71)	411.04 (275.33-602.74)	604.95 (368.17-1189.50)	407.18 (282.78-615.68)	<0.001
SIRI	1.31 (0.75-2.24)	0.81 (0.53-1.26)	1.25 (0.87-2.12)	0.81 (0.56-1.22)	<0.001

Values are presented as median (IQR) for continuous variables and n (%) for categorical variables. The training and temporal validation datasets include patients with gastric cancer and benign gastric-disease controls; p values compare gastric cancer with benign controls within the training cohort only.

^a^ Between-group comparisons were performed within the training cohort (gastric cancer vs. benign control) using the Mann–Whitney U test for continuous variables and Pearson's chi-squared test for categorical variables.

^b^ NEUT, neutrophil absolute count; LYMPH, lymphocyte absolute count; MONO, monocyte absolute count; PLT, platelet count.

^c^ NLR, neutrophil-to-lymphocyte ratio; PLR, platelet-to-lymphocyte ratio; LMR, lymphocyte-to-monocyte ratio; SII, systemic immune-inflammation index (= NEUT × PLT / LYMPH); SIRI, systemic inflammation response index (= NEUT × MONO / LYMPH). Derived inflammatory indices were not included as inputs in the primary model; they were used only for interpretation and sensitivity analyses.

### Model discrimination

Training-set five-fold cross-validated AUCs were similar across the eight algorithms (0.79–0.84) and concordant with temporal-validation discrimination (**Supplementary Table 7**), suggesting limited apparent optimism. Temporal-validation results are presented as the principal comparative evaluation.

In the temporal validation cohort, discrimination remained closely comparable across models (AUC 0.80–0.83; **Table 2**, **Figure 5**, right). The reference random-forest model achieved an AUC of 0.831 (95% CI 0.798–0.864), while XGBoost had the numerically highest AUC (0.832; 95% CI 0.800–0.865), with substantially overlapping confidence intervals. At its training-derived operating threshold (0.258, maximising the Youden index), the random forest yielded a sensitivity of 72.9%, specificity of 77.2%, positive predictive value of 50.4%, negative predictive value of 90.0%, F1 score of 0.596 and Matthews correlation coefficient of 0.450, flagging 34.8% of the validation cohort as high-risk.

**Table 2:** Temporal-validation performance (n = 735; 177 GC).

Model	AUC (95% CI)	Brier	Threshold	Sens %	Spec %	PPV %	NPV %	F1	MCC	Pred-pos %
XGBoost	0.832 (0.800–0.865)	0.134	0.188	81.9	72.9	49.0	92.7	0.613	0.478	40.3
Random forest	0.831 (0.798–0.864)	0.133	0.258	72.9	77.2	50.4	90.0	0.596	0.450	34.8
LightGBM	0.830 (0.796–0.863)	0.135	0.139	85.9	60.6	40.9	93.1	0.554	0.397	50.6
Logistic regression	0.825 (0.790–0.859)	0.135	0.190	79.7	71.9	47.3	91.8	0.594	0.449	40.5
SVM	0.824 (0.790–0.858)	0.139	0.226	67.8	78.1	49.6	88.4	0.573	0.418	32.9
k-nearest neighbours	0.823 (0.787–0.858)	0.135	0.226	71.8	78.5	51.4	89.8	0.599	0.455	33.6
Naive Bayes	0.816 (0.783–0.850)	0.154	0.142	79.1	71.7	47.0	91.5	0.589	0.442	40.5
Decision tree	0.802 (0.766–0.838)	0.146	0.243	68.4	80.1	52.2	88.9	0.592	0.446	31.6

AUC values are shown with 95% CIs estimated by DeLong's method. Thresholds are model-specific training-derived Youden points applied unchanged to the temporal validation cohort. Sens, sensitivity; Spec, specificity; PPV, positive predictive value; NPV, negative predictive value; MCC, Matthews correlation coefficient; Pred-pos, predicted-positive proportion. Brier, Brier score; lower values indicate lower overall probabilistic prediction error.

**Figure 5:**
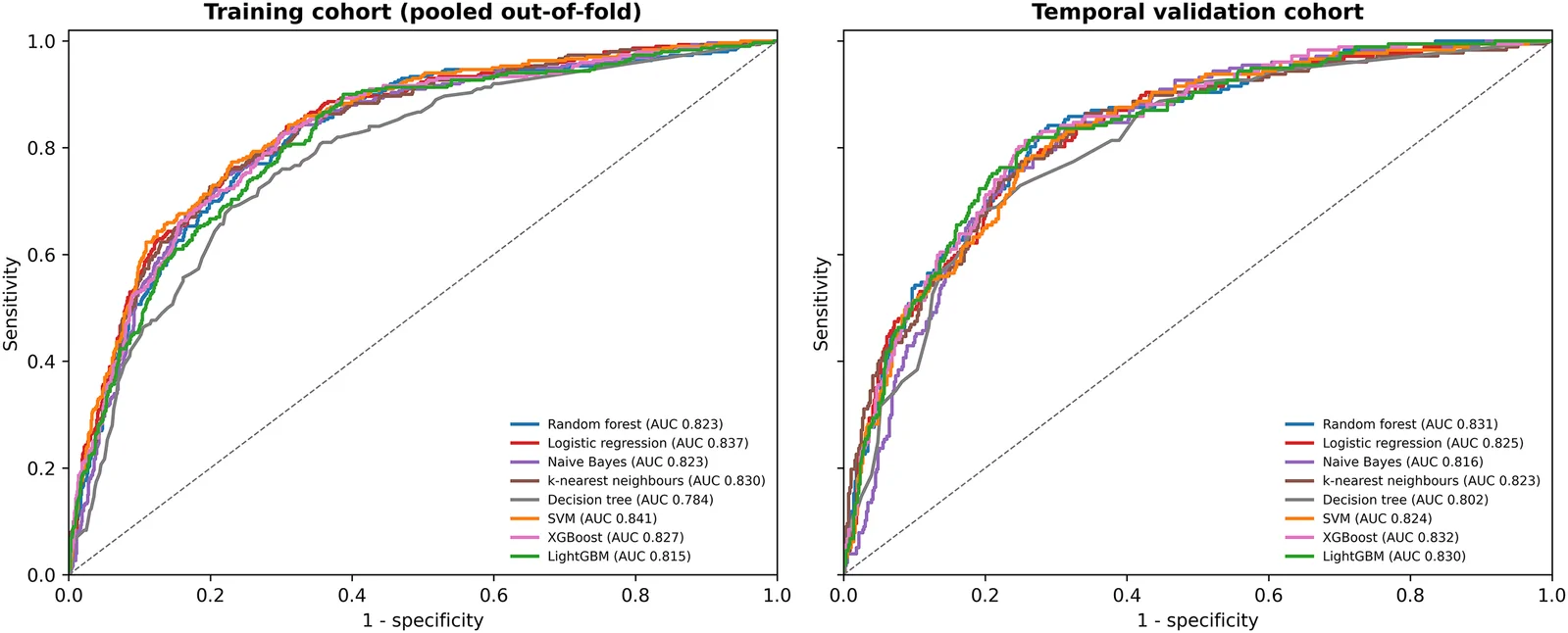
Receiver operating characteristic curves for the eight algorithms in the training cohort (pooled out-of-fold; left) and the temporal validation cohort (right). Areas under the curve are shown in the legends of each panel.

### Selection of the primary model

Because discrimination was numerically similar across algorithms, we interpreted the random forest as the reference explainable model rather than selecting a model *post hoc* on a marginal AUC difference in the temporal validation set. The random forest showed the lowest Brier score of all eight models (0.133), which is reported as a descriptive measure of overall probabilistic prediction error, and its native compatibility with exact tree-based SHAP supports transparent, individual-level explanation. Accordingly, random-forest results are used for explanation and subgroup analyses, with all algorithms reported for benchmarking.

### Calibration and clinical utility

Calibration curves based on 10 quantile bins in the temporal validation cohort lay broadly close to the diagonal for the random forest, without marked systematic over- or under-estimation of risk, and the model had a low Brier score as reported in **Table 2** (**Figure 6**). Decision-curve analysis showed positive net benefit for the model across an evaluated range of threshold probabilities (approximately 0.05–0.60), exceeding both the treat-all and treat-none strategies throughout this range in the temporal validation cohort (**Figure 7**) (46).

**Figure 6:**
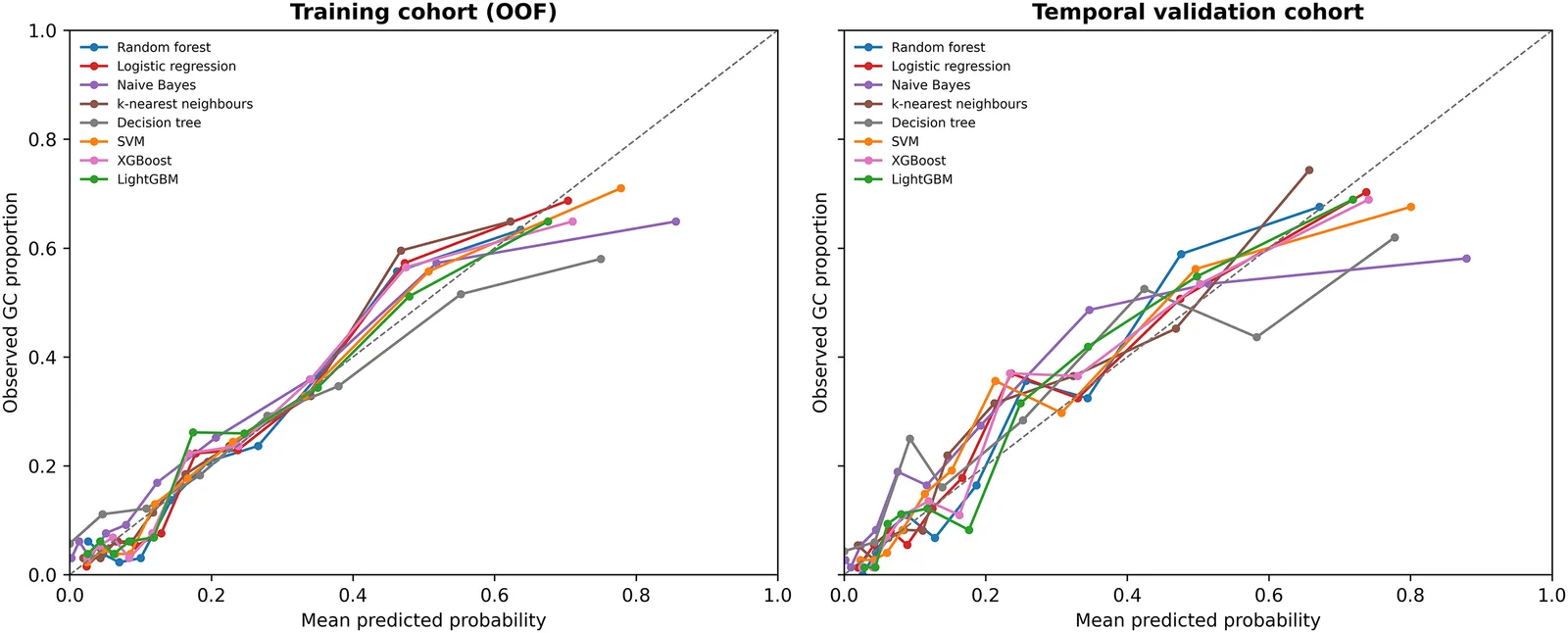
Calibration curves (observed versus mean predicted probabilities using 10 quantile bins) for the eight algorithms in the training (out-of-fold; left) and temporal validation (right) cohorts; the diagonal line denotes perfect calibration.

**Figure 7:**
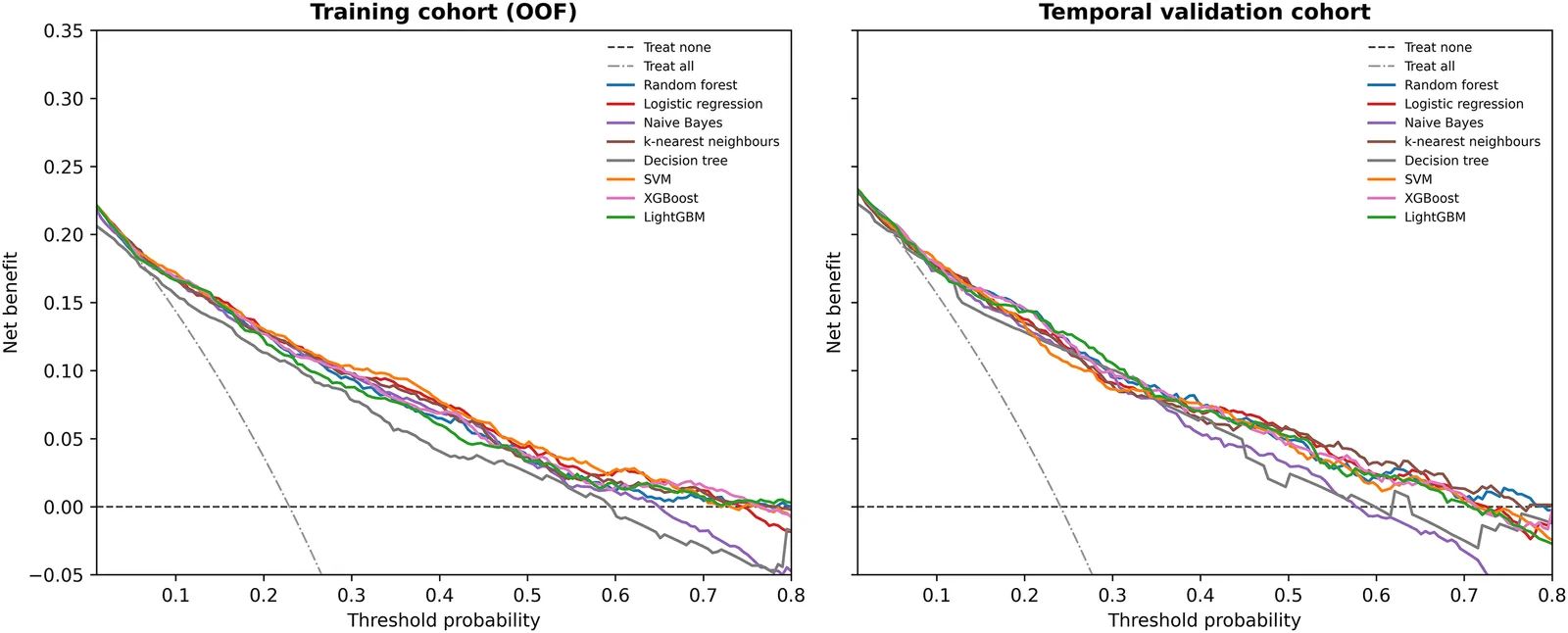
Decision-curve analysis showing net benefit across threshold probabilities for the eight algorithms in the training cohort (out-of-fold; left) and temporal validation (right) cohorts, referenced against the treat-all and treat-none strategies.

### Model explainability

Global SHAP analysis of the random forest identified the lymphocyte count as the most influential predictor (mean |SHAP| 0.086), followed by male sex (0.082), age (0.070), platelet count (0.047), monocyte count (0.033) and neutrophil count (0.018) (**Figure 2A**; **Supplementary Table 5**). The direction of these contributions was clinically plausible (**Figures 2B**, **3**): lower lymphocyte counts, male sex, older age and higher platelet and monocyte counts each increased the predicted probability of GC, while the neutrophil contribution followed a U-shaped pattern. Individual-level attributions for representative correctly classified temporal-validation cases (a GC case with predicted probability 0.747 and a benign case with 0.150) showed feature contributions consistent with the global patterns (**Figure 4**) (47).

The explanation was consistent across the two tree-based model families: an XGBoost model trained on the same six predictors yielded an identical ranking of global SHAP importance (Spearman rank correlation of mean absolute SHAP values = 1.00; **Supplementary Table 5**; **Supplementary Figure S2**).

### Model recall in tumour-marker-negative GC

Among temporal-validation GC patients in whom CEA, CA19–9 and CA72–4 had all been measured (n = 131), 45% (n = 59) were triple-negative — that is, all three markers were below their reference thresholds. Applied at its Youden operating threshold, the random forest flagged 62.7% (95% CI 50.0–73.9) of these tumour-marker-negative GC cases as high-risk, compared with 80.6% (95% CI 70.0–88.0) of the any-marker-positive GC cases (**Table 3**). Recall in the triple-negative subgroup declined with stricter thresholds (54.2% at 0.30, 40.7% at 0.40, 28.8% at 0.50). Corresponding estimates in the whole marker-measured GC cohort were concordant and are reported descriptively in **Supplementary Table 8** (triple-negative recall 68.1%, 95% CI 60.6–74.8). These results indicate that, among confirmed GC cases with measured tumour markers, the CBC model assigned high-risk probabilities to a substantial subset of triple-negative cases, although — because marker data were unavailable for benign controls — this analysis quantifies within-GC sensitivity rather than a head-to-head comparison against serology.

**Table 3:** Model recall in tumour-marker-negative GC cases (random forest).

Subgroup	n	Recall @ Youden (0.258)	@ 0.30	@ 0.40	@ 0.50
All marker-measured GC	131	72.5 (64.3–79.4)	62.6 (54.1–70.4)	50.4 (41.9–58.8)	35.1 (27.5–43.6)
Triple-negative GC	59	62.7 (50.0–73.9)	54.2 (41.7–66.3)	40.7 (29.1–53.4)	28.8 (18.8–41.4)
Any-marker-positive GC	72	80.6 (70.0–88.0)	69.4 (58.0–78.9)	58.3 (46.8–69.0)	40.3 (29.7–51.8)

Primary analysis – temporal validation cohort (out-of-sample). Denominator: GC patients with CEA, CA19–9 and CA72–4 all measured. Recall = proportion of GC patients flagged high-risk by the random-forest model; values are % (Wilson 95% CI).

Triple-negative was defined as CEA < 5 ng/mL, CA19-9 < 37 U/mL and CA72-4 < 6.9 U/mL; censored high values were treated as above threshold. The Youden threshold (0.258) is the training-derived random-forest probability cutoff.

### Sensitivity analyses

Discrimination was not improved by augmenting the six-variable model with the five derived inflammatory indices: the ratio-augmented eleven-feature model (temporal AUC 0.821) and the LASSO-selected eight-feature subset (0.820) were, if anything, marginally lower than the six-variable primary model (0.831), with no statistically significant difference (ΔAUC −0.010, DeLong p = 0.139; and −0.011, p = 0.119, respectively; **Supplementary Tables 2**, **3**). Class weighting did not improve discrimination (balanced AUC 0.830 vs 0.831; ΔAUC −0.001, p = 0.77) and increased the Brier score (0.149 vs 0.133), supporting the use of the unweighted model (**Supplementary Table 4**). Together, these analyses supported retention of the parsimonious six-variable CBC-plus-demographic specification.

## Discussion

Using only pretreatment routine CBC parameters together with age and sex, a parsimonious explainable model discriminated newly diagnosed GC from benign gastric disease, achieving an AUC of 0.831 in temporal validation, broadly acceptable graphical calibration, a low Brier score and substantial within-GC recall among tumour-marker-negative cases. Three features of the study distinguish it from previous CBC-based and laboratory-data-based cancer prediction models (15, 25–33, 38, 49–57).

First, treatment-naïve sampling and comorbidity review reduce contamination from treatment-related haematological changes and major CBC-altering disorders. Because every GC case was pathologically confirmed, newly diagnosed and treatment-naïve, with the CBC obtained before any surgery, chemotherapy or radiotherapy, and because conditions that materially perturb the blood count were excluded from both cohorts, the model inputs are less likely to be contaminated by the post-treatment inflammatory changes or competing haematological disorders that confound cohorts assembled from unverified diagnosis records. The dominant contribution of the lymphocyte count, followed by demographic factors and the platelet count, is consistent with a disease-associated peripheral-blood pattern, but residual influences from age, sex, tumour stage or bleeding, nutritional status, smoking and alcohol exposure, Helicobacter pylori status, medications, unrecorded infection and other unmeasured confounders cannot be excluded (16–24, 58–62).

Second, the parsimonious input set showed stable performance across the prespecified sensitivity analyses. Augmenting the six raw variables with five derived inflammatory indices did not improve and marginally reduced temporal discrimination, while the feature-importance ranking was identical in a second gradient-boosting model (Spearman rank correlation = 1.00). Because the required inputs come from a single ubiquitous test plus demographic data available at first contact, the model has input requirements compatible with future evaluation for opportunistic triage in primary care and low-resource settings, where endoscopic and serological capacity is limited. We regard this as a complement to, not a replacement for, existing pathways: a low-cost pre-endoscopic risk flag rather than a diagnostic test (15, 25–33, 38, 49–57).

Third, in confirmed GC cases with negative conventional serum tumour markers, the model still flagged a substantial proportion as high-risk. Nearly half of the marker-measured GC cases in temporal validation were triple-negative for CEA, CA19–9 and CA72-4, and 62.7% of these cases were assigned high-risk probabilities by the CBC model. This suggests that CBC-based risk scores may provide information not captured by marker positivity, while not constituting a direct comparison with marker testing because controls lacked marker data (8–10).

Several choices warrant comment. Discrimination was numerically similar across the eight algorithms evaluated. Rather than selecting the numerically highest model on the temporal set, which would risk optimistic, test-set-driven selection, we interpreted the random forest as the reference explainable model on methodological and interpretability grounds. Its lower Brier score is reported as a descriptive measure of overall probabilistic prediction error, not as a stand-alone proof of superior calibration or as a temporal-validation model-selection criterion. No *post-hoc* probability recalibration was applied. Class weighting was evaluated separately and degraded probabilistic accuracy without improving discrimination. The positive predictive value at the operating threshold reflects the GC proportion and case mix of the study cohort and should be interpreted accordingly when transporting the threshold to populations with different pre-test probabilities (45, 46).

Explainability is particularly relevant to the intended pre-endoscopic triage setting. Conventional regression provides interpretable population-level associations under prespecified functional forms, whereas tree-based SHAP additionally decomposes each individual prediction into the direction and magnitude of the contribution from each predictor. In a future clinical implementation, the model could therefore present not only an estimated GC probability and risk category, but also the principal factors increasing or decreasing that probability. For example, clinicians could determine whether a high-risk classification is supported by the disease-associated pattern observed in this study, such as a lower lymphocyte count together with higher platelet or monocyte counts, or whether it is dominated by an isolated or clinically unexpected laboratory value that warrants verification. These patient-level explanations would allow the model output to be interpreted alongside symptoms, comorbidities, medications and other clinical information before deciding whether to recommend endoscopy, repeat testing or routine follow-up. This traceability may enhance clinician acceptance by making predictions open to verification, challenge, documentation and communication, rather than requiring reliance on an opaque risk score. It also supports a human-in-the-loop workflow in which the model prioritises patients for further assessment while the clinician retains final decision-making authority. Nevertheless, a plausible SHAP explanation does not establish causality or guarantee that a prediction is correct; prospective usability studies, external validation, calibration assessment and clinical-impact evaluation remain necessary before implementation (32, 38, 47).

Our temporal-validation results showed that discrimination could be retained with only six pretreatment CBC and demographic variables (AUC = 0.831), with lymphocyte count, sex, age and platelet count making the largest contributions in the SHAP analysis. Recent studies help position these findings across a continuum of artificial-intelligence applications in gastric cancer. Seo et al. provide the closest clinical comparator: their model repurposed 24 routine blood-test variables together with age and sex to stratify patients with chronic dyspepsia for opportunistic endoscopy referral (15). Although differences in population, predictors and outcome definition preclude direct performance comparison, their findings and ours provide convergent evidence that routinely collected blood data can support pre-endoscopic risk stratification. Our results further suggest that useful temporal discrimination may be achievable with a substantially more parsimonious input set, while providing patient-level explanations of the principal haematological and demographic contributions. Chen et al. addressed a different, later decision point by integrating CT radiomics with clinical information to predict occult peritoneal metastasis in locally advanced gastric cancer (34). Their multicentre and prospective validation illustrates how richer multimodal information may refine management after initial case identification. Wang et al. focused on prognosis and tumour biology, integrating multidimensional omics with deep learning to identify a macrophage–fibroblast–tumour co-infiltration pattern (35). This study does not directly validate our CBC predictors but demonstrates biological heterogeneity that peripheral blood-cell counts alone cannot resolve and suggests a direction for investigating the biological basis of the haematological risk signal observed here. Collectively, these studies support complementary roles for artificial intelligence across the gastric-cancer pathway: accessible blood-based triage, imaging-assisted staging and molecularly informed prognostic stratification. The present study contributes specifically to the first of these roles by prioritising pretreatment sampling, patient-level label integrity, temporal validation and individual-level interpretability.

Related AI studies integrating metabolomic profiling or chemical-structure-aware graph learning in other diseases illustrate how high-dimensional biological data can yield diagnostically or mechanistically informative signatures, although they do not establish incremental value for gastric-cancer prediction (36, 37). In the present cohort, additional inflammatory, radiological and molecular data were not systematically available and were therefore not evaluated as model inputs. Nevertheless, these modalities could provide predictive information complementary to the current six-variable model. Circulating inflammatory mediators may reflect systemic inflammatory activity not fully captured by peripheral blood-cell counts; radiological features may characterise lesion morphology, tumour burden and patterns of local or metastatic spread; and molecular, transcriptomic or metabolomic signatures may capture intrinsic tumour biology and microenvironmental heterogeneity. Their incremental value may be greatest among patients whose CBC-derived risk estimates are intermediate or discordant with clinical assessment. However, adding higher-cost or less routinely available variables would not necessarily improve transportable clinical performance and could increase missingness, overfitting, workflow complexity and inequity of access. Future studies should therefore compare the six-variable model with nested extensions incorporating each additional modality, first within prospectively assembled cohorts in which all candidate predictors are measured in the same participants and subsequently in independent external cohorts. Any improvement should be evaluated not only by changes in AUC, but also by calibration, threshold-specific performance, decision-curve net benefit, transportability and cost-effectiveness. A staged strategy in which inexpensive CBC-based triage is followed by targeted imaging or molecular assessment among selected patients may provide a practical balance between accessibility and predictive refinement.

This study has several limitations. First, the retrospective design necessarily relied on information available in existing clinical records. Although all retained GC cases were pathologically confirmed, newly diagnosed, treatment-naïve and reviewed at the individual-case level, benign and outpatient classifications were based primarily on clinician-assigned diagnoses. Consequently, occult or early GC may have been misclassified as benign, although such misclassification would generally be expected to attenuate rather than inflate discrimination. Second, restriction to patients with an eligible baseline CBC may limit applicability to clinical encounters in which CBC testing is unavailable at the index assessment. An age-and-sex-only comparator was not evaluated; therefore, the incremental predictive contribution of the four CBC variables beyond demographic characteristics was not quantified. Third, because tumour markers were not routinely measured in benign controls, the marker-negative analysis estimates sensitivity within confirmed GC rather than providing a head-to-head comparison with serological testing. The temporal-validation triple-negative subgroup was also modest in size, resulting in wide confidence intervals. Histological composition and subtype-specific performance could not be characterised, and common chronic conditions were retained to preserve real-world applicability, although their potential residual influence on CBC measurements cannot be completely excluded. Finally, and most importantly, temporal validation within a single institution assesses model stability across time but does not establish geographic or institutional generalisability. Differences among institutions in patient case mix, GC prevalence, referral practices, diagnostic coding, CBC analysers, laboratory reference ranges and pre-analytical procedures may alter predicted-risk distributions, calibration and threshold-specific performance, even when discrimination remains relatively stable. Prospective and independent external validation across multiple hospitals, geographic regions and levels of care is therefore an essential prerequisite before clinical implementation. Such studies should quantify between-centre heterogeneity and assess discrimination, calibration, transportability of prespecified thresholds, decision-curve net benefit, workflow feasibility, clinical impact and cost-effectiveness, as well as whether local recalibration or threshold adjustment is required. Until this evidence is available, the present model should be regarded as a candidate triage approach requiring further validation rather than a tool ready for routine clinical use (38, 39).

## Conclusions

A parsimonious, explainable machine-learning model combining pretreatment CBC parameters with age and sex discriminated newly diagnosed GC from benign gastric disease in a temporally separated validation cohort and showed broadly acceptable graphical calibration and a low Brier score. Restriction of the GC cohort to pathologically confirmed, newly diagnosed, treatment-naïve cases with an admission CBC obtained before treatment, together with harmonised comorbidity screening and dual-reviewer adjudication, reduced the risk of contamination from treatment-related haematological changes. The model classified a substantial proportion of tumour-marker-negative GC cases as high risk, and feature ranking was consistent in a second tree-based algorithm. Because the required inputs are routinely available, the model warrants external evaluation as a potential pre-endoscopic triage aid. Prospective multicentre validation is required before clinical implementation.

## Data Availability

The datasets analysed in this study are not publicly available because they contain potentially sensitive clinical information. Requests to access the de-identified data may be directed to the corresponding author and will be considered in accordance with applicable ethical and institutional requirements. The supporting results are included in the article and **Supplementary Material**.

## Supplementary material

Supplementary Figure 1 Spearman correlation heatmap of the eleven candidate predictors.

Supplementary Figure 2 Global SHAP importance and summary for the XGBoost model in the temporal validation cohort. This analysis was performed as an explainability-robustness check using the same six predictors as the primary random-forest model.

Supplementary Table 1 Diagnosis-keyword rules and non-index modifiers used for cohort assignment. Structured HIS/LIS diagnosis fields were screened with the predefined keyword rules below. Non-index modifiers were used to exclude non-index GC records; final cohort membership was confirmed by two independent reviewers through manual chart review (see Methods). GC, gastric cancer. Non-index modifiers included personal history, post-operative status, follow-up/surveillance review, historical record, external prior diagnosis and uncertain-diagnosis marker; these indicated records that did not represent newly diagnosed, treatment-naïve index GC and were used only for screening before final manual chart review.

Supplementary Table 2 Variance inflation factors and LASSO selection (ratio-augmented set). VIF, variance inflation factor; LASSO, least absolute shrinkage and selection operator. Post-LASSO VIF values were calculated among variables retained after LASSO selection; dash indicates variables not retained by LASSO.

Supplementary Table 3 Ratio-augmented feature-representation sensitivity analysis. CV AUC values are mean ± SD across five folds. Temporal AUC values are shown with 95% CIs estimated by DeLong's method; ΔAUC and DeLong p values are versus the six-variable primary model. Brier, Brier score; lower values indicate lower overall probabilistic prediction error.

Supplementary Table 4 Class-weighting sensitivity analysis (random forest). Class weighting was applied during model training only. Temporal AUC values are shown with 95% CIs estimated by DeLong's method; ΔAUC and DeLong p values are versus the unweighted random-forest primary model. Brier, Brier score; lower values indicate lower overall probabilistic prediction error.

Supplementary Table 5 Cross-model global feature importance (explainability robustness). Mean absolute SHAP values are shown for the primary random forest and an XGBoost model trained on the same six predictors. Random-forest permutation importance is provided for reference. Features are ordered by random-forest mean |SHAP|; the Spearman rank correlation between random-forest and XGBoost mean |SHAP| was 1.00, indicating identical ordering of all six predictors.

Supplementary Table 6 Operating characteristics at fixed decision thresholds in temporal validation. All eight models are shown at the model-specific training-derived Youden threshold and at fixed probability thresholds of 0.30, 0.40 and 0.50. Sens, sensitivity; Spec, specificity; PPV/NPV, positive/negative predictive value; F1, F1 score; MCC, Matthews correlation coefficient; Pred-pos, predicted-positive proportion.

Supplementary Table 7 Training-set discrimination of candidate algorithms. CV AUC values are mean ± SD across five folds. OOF AUC was calculated from pooled out-of-fold predictions from the same cross-validation procedure (**Figure 5**, left).

Supplementary Table 8 Descriptive model recall in the whole marker-measured GC cohort. 1. GC, gastric cancer; CEA, carcinoembryonic antigen; CA19-9, carbohydrate antigen 19-9; CA72-4, carbohydrate antigen 72-4. Triple-negative was defined as CEA <5 ng/mL, CA19-9 <37 U/mL and CA72-4 <6.9 U/mL; censored high values were treated as above threshold. 2. Descriptive analysis was performed in the whole marker-measured GC cohort (training + temporal; includes in-sample cases). The denominator was GC patients with CEA, CA19-9 and CA72-4 all measured. Values are recall percentages with Wilson 95% CIs. 3. Recall was calculated as the proportion of patients flagged high-risk by the primary random-forest model. The Youden threshold (0.258) is the training-derived probability cutoff of the primary random-forest model.

Supplementary Table 9 Hyperparameter tuning grid and selected settings for candidate algorithms. Hyperparameter tuning was performed within the training cohort only by five-fold cross-validation, with AUC as the optimisation criterion. The temporal validation cohort was not used for feature handling, hyperparameter tuning, threshold selection or model fitting. Scaling was applied only to algorithms that require or benefit from standardisation.
