# Supplementary material for: Explainable machine learning using pretreatment complete blood count parameters and demographic characteristics for gastric cancer risk stratification: a temporal validation study
Source: Front Immunol. 2026 Oct 15;17:1892111. doi: 10.3389/fimmu.2026.1892111 (PMC13716773; doi:10.3389/fimmu.2026.1892111)
Supplement: Supplementary file 1 [file SupplementaryFile1.pdf]

Supplementary material

Supplementary Table S1. Diagnosis-keyword rules and non-index modifiers used for cohort assignment

Structured HIS/LIS diagnosis fields were screened with the predefined keyword rules below. Non-index modifiers were used to exclude non-index GC records; final cohort membership was confirmed by two independent reviewers through manual chart review (see Methods).

Section	#	Diagnosis keyword
**GC diagnosis keywords**	1	Gastric malignancy
	2	Gastro-oesophageal junction malignancy
	3	Cardia–corpus malignancy
	4	Antral malignancy
	5	Corpus malignancy
	6	Remnant stomach malignancy
	7	Cardia–fundus malignancy
	8	Fundal malignancy
**Benign gastric-disease keywords**	1	Chronic gastritis
	2	Chronic superficial gastritis
	3	Erosive gastritis
	4	Reflux gastritis
	5	Bile reflux gastritis
	6	Verrucous gastritis
	7	Gastric polyps (including multiple gastric polyps)
	8	Gastric ulcer (with or without bleeding)
	9	Remnant gastritis
	10	Eosinophilic gastritis

GC, gastric cancer. Non-index modifiers included personal history, post-operative status, follow-up/surveillance review, historical record, external prior diagnosis and uncertain-diagnosis marker; these indicated records that did not represent newly diagnosed, treatment-naïve index GC and were used only for screening before final manual chart review.

Supplementary Table S2. Variance inflation factors and LASSO selection (ratio-augmented set)

Feature	VIF (11-feature)	VIF (post-LASSO)	LASSO coef	Retained
age_years	1.11	1.10	0.597	Yes
sex_male	1.11	1.11	0.594	Yes
NEUT	5.87	4.04	-0.236	Yes
LYMPH	7.22	3.88	-0.306	Yes
MONO	11.30	—	0.000	No
PLT	4.86	3.53	0.439	Yes
NLR	15.56	—	0.000	No
PLR	16.70	14.65	0.123	Yes
LMR	7.32	1.95	-0.649	Yes
SII	15.31	11.91	0.076	Yes
SIRI	15.78	—	0.000	No

VIF, variance inflation factor; LASSO, least absolute shrinkage and selection operator. Post-LASSO VIF values were calculated among variables retained after LASSO selection; dash indicates variables not retained by LASSO.

Supplementary Table S3. Ratio-augmented feature-representation sensitivity analysis

Feature set	k	CV AUC ± SD	Temporal AUC (95% CI)	ΔAUC vs primary	DeLong p	Brier
6-variable primary	6	0.827 ± 0.026	0.831 (0.798–0.864)	+0.000	—	0.133
+ ratios (11 features)	11	0.828 ± 0.027	0.821 (0.787–0.855)	-0.010	0.139	0.140
LASSO-selected subset	8	0.828 ± 0.028	0.820 (0.786–0.854)	-0.011	0.119	0.140

CV AUC values are mean ± SD across five folds. Temporal AUC values are shown with 95% CIs estimated by DeLong's method; ΔAUC and DeLong p values are versus the six-variable primary model. Brier, Brier score; lower values indicate lower overall probabilistic prediction error.

Supplementary Table S4. Class-weighting sensitivity analysis (random forest)

Model	Class weight	Temporal AUC (95% CI)	Brier	ΔAUC	DeLong p
Random forest primary	—	0.831 (0.798–0.864)	0.133	+0.000	—
Random forest class-weighted	balanced	0.830 (0.797–0.864)	0.149	-0.001	0.767

Class weighting was applied during model training only. Temporal AUC values are shown with 95% CIs estimated by DeLong's method; ΔAUC and DeLong p values are versus the unweighted random-forest primary model. Brier, Brier score; lower values indicate lower overall probabilistic prediction error.

Supplementary Table S5. Cross-model global feature importance (explainability robustness)

Feature	RF mean |SHAP|	XGBoost mean |SHAP|	RF permutation importance
LYMPH	0.086	0.096	0.062
sex (male)	0.082	0.074	0.083
age	0.070	0.072	0.082
PLT	0.047	0.063	0.042
MONO	0.033	0.043	0.020
NEUT	0.018	0.025	0.007

Mean absolute SHAP values are shown for the primary random forest and an XGBoost model trained on the same six predictors. Random-forest permutation importance is provided for reference. Features are ordered by

random-forest mean |SHAP|; the Spearman rank correlation between random-forest and XGBoost mean |SHAP| was 1.00, indicating identical ordering of all six predictors.

Supplementary Table S6. Operating characteristics at fixed decision thresholds in temporal validation

Model	Threshold	Sens %	Spec %	PPV %	NPV %	F1	MCC	Pred-pos %
Random forest	Youden (0.258)	72.9	77.2	50.4	90.0	0.596	0.450	34.8
	0.30	63.8	82.1	53.1	87.7	0.579	0.433	29.0
	0.40	51.4	90.3	62.8	85.4	0.565	0.448	19.7
	0.50	38.4	94.3	68.0	82.8	0.491	0.408	13.6
XGBoost	Youden (0.188)	81.9	72.9	49.0	92.7	0.613	0.478	40.3
	0.30	63.8	83.7	55.4	87.9	0.593	0.454	27.8
	0.40	50.8	90.0	61.6	85.2	0.557	0.437	19.9
	0.50	41.2	92.8	64.6	83.3	0.503	0.404	15.4
LightGBM	Youden (0.139)	85.9	60.6	40.9	93.1	0.554	0.397	50.6
	0.30	67.8	82.4	55.0	89.0	0.608	0.470	29.7
	0.40	51.4	89.2	60.3	85.3	0.555	0.430	20.5
	0.50	41.8	93.5	67.3	83.5	0.516	0.424	15.0
Logistic regression	Youden (0.190)	79.7	71.9	47.3	91.8	0.594	0.449	40.5
	0.30	62.1	82.1	52.4	87.2	0.568	0.419	28.6
	0.40	49.7	90.3	62.0	85.0	0.552	0.434	19.3
	0.50	40.1	94.8	71.0	83.3	0.513	0.435	13.6
SVM	Youden (0.226)	67.8	78.1	49.6	88.4	0.573	0.418	32.9
	0.30	56.5	84.9	54.3	86.0	0.554	0.409	25.0
	0.40	50.3	91.0	64.0	85.2	0.563	0.451	18.9
	0.50	42.9	93.2	66.7	83.7	0.522	0.427	15.5
k-nearest neighbours	Youden (0.226)	71.8	78.5	51.4	89.8	0.599	0.455	33.6
	0.30	59.3	83.0	52.5	86.5	0.557	0.406	27.2
	0.40	48.6	89.4	59.3	84.6	0.534	0.408	19.7
	0.50	39.5	95.0	71.4	83.2	0.509	0.434	13.3
Naive Bayes	Youden (0.142)	79.1	71.7	47.0	91.5	0.589	0.442	40.5
	0.30	62.7	82.8	53.6	87.5	0.578	0.433	28.2
	0.40	49.7	86.9	54.7	84.5	0.521	0.379	21.9
	0.50	39.0	91.6	59.5	82.6	0.471	0.358	15.8
Decision tree	Youden (0.243)	68.4	80.1	52.2	88.9	0.592	0.446	31.6
	0.30	68.4	80.1	52.2	88.9	0.592	0.446	31.6
	0.40	58.8	84.4	54.5	86.6	0.565	0.421	26.0
	0.50	38.4	89.6	54.0	82.1	0.449	0.318	17.1

All eight models are shown at the model-specific training-derived Youden threshold and at fixed probability thresholds of 0.30, 0.40 and 0.50. Sens, sensitivity; Spec, specificity; PPV/NPV, positive/negative predictive value; F1, F1 score; MCC, Matthews correlation coefficient; Pred-pos, predicted-positive proportion.

Supplementary Table S7. Training-set discrimination of candidate algorithms

Model	Mean CV AUC ± SD	OOF AUC
SVM	0.842 ± 0.022	0.841
Logistic regression	0.839 ± 0.027	0.837
k-nearest neighbours	0.832 ± 0.021	0.830
XGBoost	0.830 ± 0.029	0.827
Random forest	0.827 ± 0.026	0.823
Naive Bayes	0.825 ± 0.023	0.823
LightGBM	0.820 ± 0.035	0.815
Decision tree	0.785 ± 0.025	0.784

CV AUC values are mean ± SD across five folds. OOF AUC was calculated from pooled out-of-fold predictions from the same cross-validation procedure (Figure 5, left).

Supplementary Table S8. Descriptive model recall in the whole marker-measured GC cohort

Subgroup	n	Recall @ Youden (0.258)	@ 0.30	@ 0.40	@ 0.50
All marker-measured GC	341	73.0 (68.1–77.5)	64.8 (59.6–69.7)	49.3 (44.0–54.6)	32.3 (27.5–37.4)
Triple-negative GC	160	68.1 (60.6–74.8)	60.0 (52.3–67.3)	46.2 (38.7–54.0)	32.5 (25.7–40.1)
Any-marker-positive GC	181	77.3 (70.7–82.8)	69.1 (62.0–75.3)	51.9 (44.7–59.1)	32.0 (25.7–39.2)

1. GC, gastric cancer; CEA, carcinoembryonic antigen; CA19-9, carbohydrate antigen 19-9; CA72-4, carbohydrate antigen 72-4. Triple-negative was defined as CEA <5 ng/mL, CA19-9 <37 U/mL and CA72-4 <6.9 U/mL; censored high values were treated as above threshold.
2. Descriptive analysis was performed in the whole marker-measured GC cohort (training + temporal; includes in-sample cases). The denominator was GC patients with CEA, CA19-9 and CA72-4 all measured. Values are recall percentages with Wilson 95% CIs.
3. Recall was calculated as the proportion of patients flagged high-risk by the primary random-forest model. The Youden threshold (0.258) is the training-derived probability cutoff of the primary random-forest model.

Supplementary Table S9. Hyperparameter tuning grid and selected settings for candidate algorithms

Model	Standardised before model	Tuned hyperparameters	Values explored	Best five-fold CV AUC	Selected hyperparameters
Random forest	No	n_estimators fixed at 500; max_depth; min_samples_leaf; max_features	max_depth: 4, 6, 8, None; min_samples_leaf: 5, 10, 20; max_features: sqrt, None	0.827	{"model__max_depth": null, "model__max_features": "sqrt", "model__min_samples_leaf": 10}
Logistic regression	Yes	C	C: 0.01, 0.1, 1.0, 10.0	0.839	{"model__C": 0.1}
Naive Bayes	Yes	var_smoothing	var_smoothing: 1e-10, 1e-9, 1e-8, 1e-7	0.825	{"model__var_smoothing": 1e-10}
k-nearest neighbours	Yes	n_neighbors; weights	n_neighbors: 5, 11, 21, 31, 51; weights: uniform, distance	0.832	{"model__n_neighbors": 51, "model__weights": "distance"}
Decision tree	No	max_depth; min_samples_leaf	max_depth: 2, 3, 4, 5, None; min_samples_leaf: 5, 10, 20, 40	0.785	{"model__max_depth": null, "model__min_samples_leaf": 20}

SVM	Yes	C; gamma; kernel	C: 0.1, 1.0, 10.0; gamma: scale, 0.1, 0.01; kernel: rbf	0.842	{"model__C": 1.0, "model__gamma": 0.01, "model__kernel": "rbf"}
XGBoost	No	n_estimators; max_depth; learning_rate; subsample; colsample_bytree	n_estimators: 150, 250; max_depth: 2, 3, 4; learning_rate: 0.03, 0.06; subsample: 0.8, 1.0; colsample_bytree: 0.8, 1.0	0.83	{"model__colsample_bytree": 0.8, "model__learning_rate": 0.03, "model__max_depth": 2, "model__n_estimators": 250, "model__subsample": 0.8}
LightGBM	No	n_estimators; max_depth; learning_rate; num_leaves; min_child_samples	n_estimators: 150, 250; max_depth: -1, 3, 5; learning_rate: 0.03, 0.06; num_leaves: 15, 31; min_child_samples: 10, 20	0.82	{"model__learning_rate": 0.03, "model__max_depth": 3, "model__min_child_samples": 10, "model__n_estimators": 150, "model__num_leaves": 15}

Hyperparameter tuning was performed within the training cohort only by five-fold cross-validation, with AUC as the optimisation criterion. The temporal validation cohort was not used for feature handling, hyperparameter tuning, threshold selection or model fitting. Scaling was applied only to algorithms that require or benefit from standardisation.
